# Nitric Oxide Acts as a Positive Regulator to Induce Metamorphosis of the Ascidian *Herdmania momus*

**DOI:** 10.1371/journal.pone.0072797

**Published:** 2013-09-03

**Authors:** Nobuo Ueda, Sandie M. Degnan

**Affiliations:** School of Biological Sciences, University of Queensland, Brisbane, Queensland, Australia; Ecole Normale Supérieure de Lyon, France

## Abstract

Marine invertebrates commonly have a biphasic life cycle in which the metamorphic transition from a pelagic larva to a benthic post-larva is mediated by the nitric oxide signalling pathway. Nitric oxide (NO) is synthesised by nitric oxide synthase (NOS), which is a client protein of the molecular chaperon heat shock protein 90 (HSP90). It is notable, then, that both NO and HSP90 have been implicated in regulating metamorphosis in marine invertebrates as diverse as urochordates, echinoderms, molluscs, annelids, and crustaceans. Specifically, the suppression of NOS activity by the application of either NOS- or HSP90-inhibiting pharmacological agents has been shown consistently to induce the initiation of metamorphosis, leading to the hypothesis that a negative regulatory role of NO is widely conserved in biphasic life cycles. Further, the induction of metamorphosis by heat-shock has been demonstrated for multiple species. Here, we investigate the regulatory role of NO in induction of metamorphosis of the solitary tropical ascidian, *Herdmania momus*. By coupling pharmacological treatments with analysis of *HmNOS* and *HmHSP90* gene expression, we present compelling evidence of a positive regulatory role for NO in metamorphosis of this species, in contrast to all existing ascidian data that supports the hypothesis of NO as a conserved negative regulator of metamorphosis. The exposure of competent *H. momus* larvae to a NOS inhibitor or an NO donor results in an up-regulation of *NOS* and *HSP90* genes. Heat shock of competent larvae induces metamorphosis in a temperature dependent manner, up to a thermal tolerance that approaches 35°C. Both larval/post-larval survival and the appearance of abnormal morphologies in *H. momus* post-larvae reflect the magnitude of up-regulation of the *HSP90* gene in response to heat-shock. The demonstrated role of NO as a positive metamorphic regulator in *H. momus* suggests the existence of inter-specific adaptations of NO regulation in ascidian metamorphosis.

## Introduction

Marine benthic communities are dominated by invertebrate animals with a biphasic life cycle that is characterised by a pelagic larval phase of variable length and a reproductive benthic adult phase [1]–[3]. The transition from larva to adult requires that the free-swimming larva makes a habitat shift to settle on to a benthic substrate, where it morphologically and physiologically metamorphoses into the benthic form [3], [4]. Generally, the initiation of settlement and metamorphosis must meet two requirements. First, the planktonic larvae must attain ontogenic maturation, known as competency [2]. Second, competent larvae of most species need to receive specific environmental cues to be induced to settle and, subsequently, to initiate metamorphosis [4]. Known inductive cues include the surface texture of substrates or waterborne chemical ligands that are released from conspecifics, microbial films, and prey species, all of which may be used by the competent larvae to assess the suitability and quality of habitats for post-metamorphic life [5]. In some species, exposure to an acute environmental stress such as a heat-shock may be sufficient to induce metamorphosis of competent larvae, even in the absence of any substrate-derived inductive cues [6]–[9]. Furthermore, some species are capable of spontaneous metamorphosis, again in the absence of any external inductive cues [10].

To perceive inductive cues from the surrounding environment, marine invertebrate larvae use sensory organs operated in concert with a larval nervous system [11]. The binding of environmental cues to cell surface receptors on the larval sensory organs transmits signals via the larval nervous system to activate biochemical signalling pathways that drive the global morphogenetic events of metamorphosis [12], [13]. Not surprisingly then, settlement and metamorphosis of many species can successfully be induced *in vitro*, by the application of synthetic chemical agents that activate or inhibit parts of these signalling pathways that are conserved among metazoans [14]–[18].

One such conserved pathway is the nitric oxide (NO) signalling pathway. Nitric oxide is a gaseous second messenger molecule that regulates numerous physiological responses in both prokaryotes and eukaryotes [19], [20]. The endogenous production of NO mainly relies on an enzyme, nitric oxide synthase (NOS), which converts a substrate, L-arginine, to L-citrulline and NO [21]. Remarkably, NO regulates the timing of life cycle transitions by coordinating the developmental state of organisms with signals from surrounding environments in a huge variety of taxa, including bacteria, fungi, slime mould, plants, and animals [22]–[27]. To maintain its normal enzymatic activity, NOS requires the molecular chaperon heat shock protein 90 (HSP90) [28]–[30]. Under normal cellular conditions, HSP90 constitutively interacts with NOS – and numerous other signalling proteins - to maintain its functional conformation [31], [32]; this can be demonstrated, for example, by the application of an HSP90-inhibiting pharmacological agent that results in the degradation of NOS conformation [33]. HSP90 is a heat-inducible protein whose function is diverted into re-folding of denatured proteins during heat-shock and other situations of cellular stress [34]. This, consequently, will decrease the constitutive chaperone activity of HSP90, leading in turn to the suppression of NOS activity [35].

It is notable, then, that both NO and HSP90 have been implicated in regulating settlement and metamorphosis in multiple taxa of marine invertebrates as diverse as urochordates, echinoderms, molluscs, annelids, and crustaceans [36]–[43]. Specifically, the suppression of NOS activity by the application of either NOS- or HSP90-inhibiting pharmacological agents has been shown to induce the settlement and metamorphosis in all of these marine invertebrates representing diverse taxa [36]–[43]. Ten years ago, based on a small number of published studies at that time, Bishop & Brandhorst [35] first speculated that a negative regulatory role of NO might be a widely conserved characteristic of bilaterians. They further speculated that it is the modulation of NO production via interactions between NOS and HSP90 that regulates the initiation of metamorphosis [35]. All relevant marine invertebrate studies published so far since that hypothesis have reported consistent results; that is, a negative regulatory role of NO in metamorphosis [36]–[43].

Nitric oxide acts as a negative (repressive) regulator of settlement and metamorphosis of competent tadpole larvae in multiple species of ascidians (phylum Chordata) [38], [39]. The application of either NOS inhibitors or HSP90 inhibitors is sufficient to initiate metamorphosis in *Boltenia villosa*, *Cnemidocarpa finmarkiensis*, and *Ciona intestinalis* [38], [39]. However, in the presence of the HSP90 inhibitors, the progress of post-larval development is impeded shortly after tail resorption, suggesting that normal HSP90 function is required for completion of metamorphosis [38]. Since HSP90 is an essential protein for normal enzymatic activity of NOS [28]–[30], and since both NOS and HSP90 inhibitors can induce metamorphosis, Bishop *et al*. [38] hypothesised that NO inhibits metamorphosis in these ascidians via the activity of NOS interacting with HSP90. Consistent with this, an application of NO donors inhibits the initiation of metamorphosis in *C. intestinalis*, providing further evidence for the negative regulatory role of NO [39]. Further, the induction of metamorphosis by heat-shock has been demonstrated for two species of ascidian - *Herdmania momus* and *C. intestinalis* [7], [44]. Because NOS is a client protein of HSP90, there is a positive correlation between NO synthesis (by NOS) and HSP90 concentration; this has been demonstrated *in vitro* [29], [33]. Reduced constitutive HSP90 chaperone activity in the face of heat-shock is thus expected to diminish NOS activity, causing the induction of metamorphosis in a manner analogous to the application of NOS-inhibiting pharmacological agents [35].

Here, we investigate the regulatory role of NO in induction of metamorphosis in the solitary ascidian, *Herdmania momus* (Chordata: Urochordata: Pyuridae), which commonly inhabits the underside of coral boulders and rocks on the reef crest of the Great Barrier Reef [45], [46]. As is typical for solitary ascidians, embryos hatch in the water column as lecithotrophic (non-feeding) tadpole larvae [47]. Larval competency is acquired by 13.5–14 hour post fertilisation (hpf) at 25°C, and settlement and metamorphosis can be efficiently induced (>90%) by the introduction of 40 mM KCl-elevated sea water [45]. *Herdmania momus* also has relatively high rates of spontaneous metamorphosis (30–40% of larvae), allowing us to investigate both inductive and inhibitory effects of external cues [45]. In addition, heat-shock induces metamorphosis of *H. momus* in a temperature-dependent manner [44].

**Figure 1:**
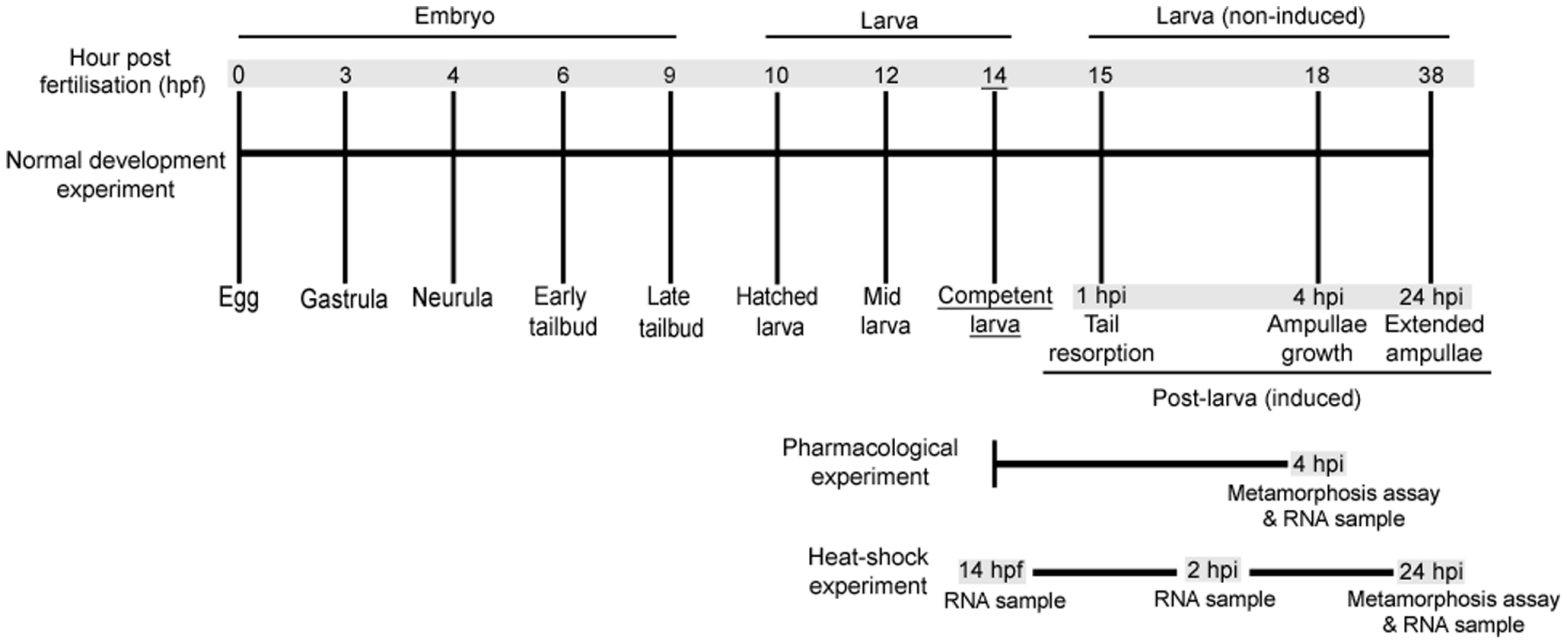
A time course of *Herdmania momus* development indicating experimental strategies employed in this study. Developmental stages are indicated by hours post fertilisation (hpf) for embryonic and larval development. Post-larval development is indicated by hours post induction (hpi). All metamorphosis assays were initiated at competency (14 hpf). Grey shading indicates times at which RNA was sampled.

Specifically, we first assess the effects of various NOS inhibitors, NO donors, and heat-shocks on the initiation of settlement and metamorphosis. These bioassays are coupled with *NOS* and *HSP90* gene expression analysis using quantitative reverse transcriptase-PCR to examine 1) the temporal profile of *NOS* and *HSP90* expression through embryonic, larval, and post-larval development, 2) the effects of NOS inhibitors and NO donors on *NOS* and *HSP90* expression at metamorphosis, and 3) the effects of the different heat-shock temperatures on *NOS* and *HSP90* expression at metamorphosis. A time-course schematic of *H. momus* development, indicating our experimental sampling points, is presented in Figure 1.

## Results

### NO is a Positive Regulator of *H. momus* Metamorphosis

Pharmacological experiments using both NOS inhibitors and NO donors (Table 1) clearly demonstrate that NO induces metamorphosis of *H. momus* larvae by 4 hour post induction (hpi). In contrast to expectations based on previously published data from other ascidian species, our results thus provide strong evidence that NO acts as a positive, rather than a negative, regulator of metamorphosis in this species.

**Table 1:** Summary of chemicals and their concentrations used in metamorphosis assay of *H. momus*.

Function	Pharmacological agents	Concentrations (mM)	Citation(s)
**NOS inhibitors**	L-NAME (L-nitroarginine methyl ester)	0.01, 0.1, 1, & 10	Froggett & Leise (1999) Pechenik *et al*. (2007) Bishop *et al*. (2008)
	AGH (aminoguanidine hemisulfate)	0.1, 0.5, & 1	Pechenik *et al*. (2007) Biggers *et al*. (2011) Zhang *et al*. (2012)
	SMIS (S-methylisothiourea sulphate)	0.05, 0.1, & 0.5	Pechenik *et al*. (2007) Biggers *et al*. (2011) Zhang *et al*. (2012)
**NO donors**	SNAP (S-nitroso-N-acetyl-penicillamine)	0.01, 0.1, & 1	Froggett & Leise (1999) Bishop *et al*. (2008)
	L-Arginine	0.01, 0.1, & 1	Bishop *et al*. (2008)
	nor-NOHA (N-hydroxy-nor-arginine)	0.1, 0.3, & 1	Comes *et al*. (2007)

**Figure 2:**
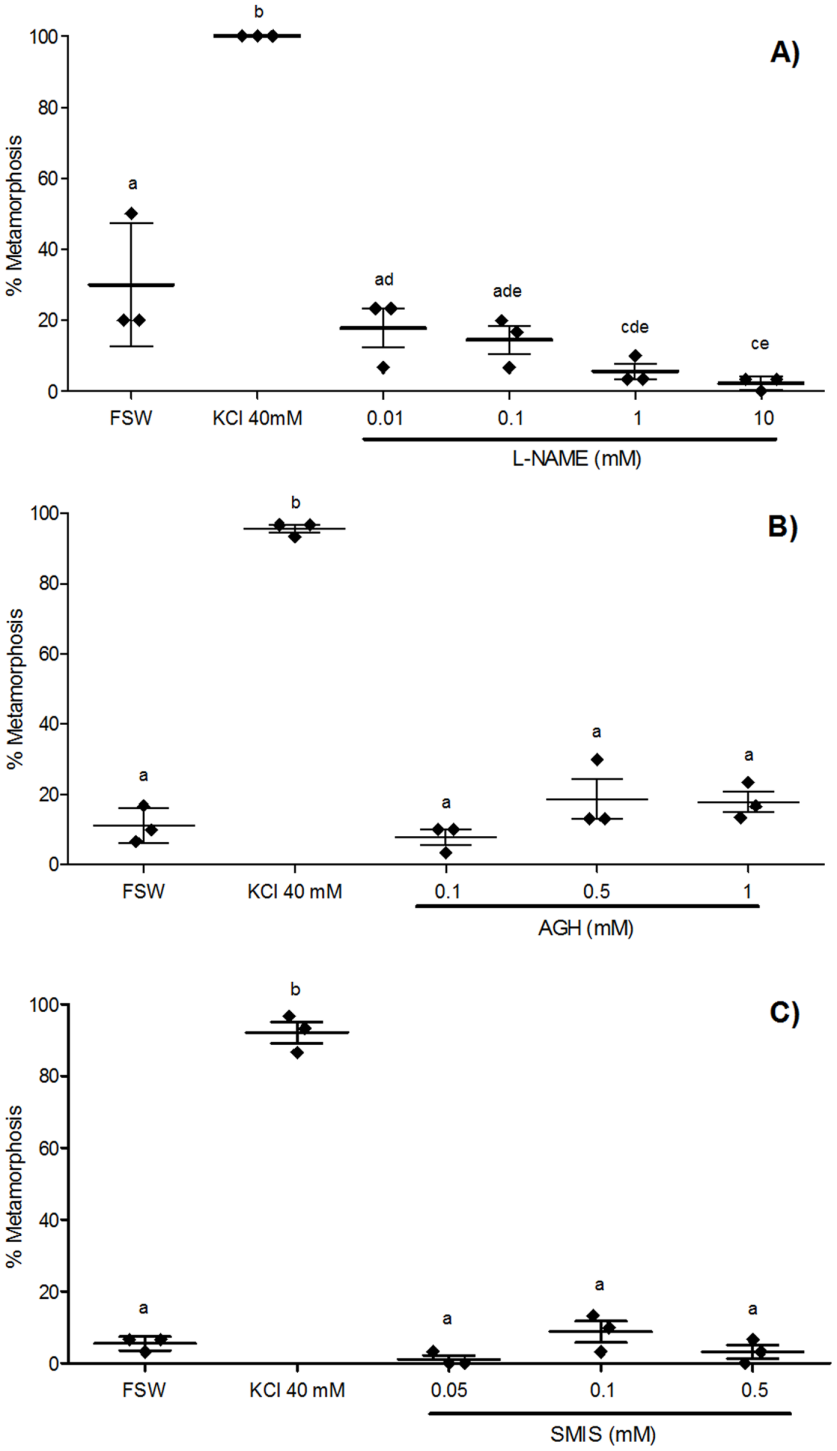
Effect of NOS inhibitors on metamorphosis of *Herdmania momus.* (A) L-nitroarginine methyl ester (L-NAME), (B) aminoguanidine hemisulfate (AGH), and (C) S-methylisothiourea sulphate (SMIS) were applied at various concentrations to competent larvae (14 hpf). The number of individuals undergoing metamorphosis was counted 4 h after the initiation of exposure (4 hpi). Filtered sea water (FSW) and 40 mM KCl-elevated FSW were used as negative and positive controls, respectively. Data are presented as the mean percentage of larval metamorphosis ± SEM (n = 3 for each treatment, 30 larvae per replicate). Diamonds indicate the actual percentages of larval metamorphosis in each replicate. Letters above error bars indicate statistically significant differences (P<0.05), as determined by one-way analysis of variance and Tukey’s HSD *post hoc* testing.

In the experiment using L-NAME as a NOS inhibitor, the higher concentrations (1 and 10 mM) significantly inhibited larval metamorphosis compared with filtered seawater (FSW) controls (Fig. 2A). Neither of the other NOS inhibitors used in this study (AGH, SMIS) produced significant effects at any of the concentrations used compared with FSW controls (Fig. 2B, C). However, given the rates of spontaneous metamorphosis were below 15% in these two experiments, the efficacy of inhibition would need to be almost complete to detect a significant difference. Importantly, in all three experiments, the positive control of KCl 40 mM induced significantly higher mean percentages of larval metamorphosis compared with any other treatments, demonstrating that inhibitors of NOS do not induce metamorphosis (Fig. 2A–C).

**Figure 3:**
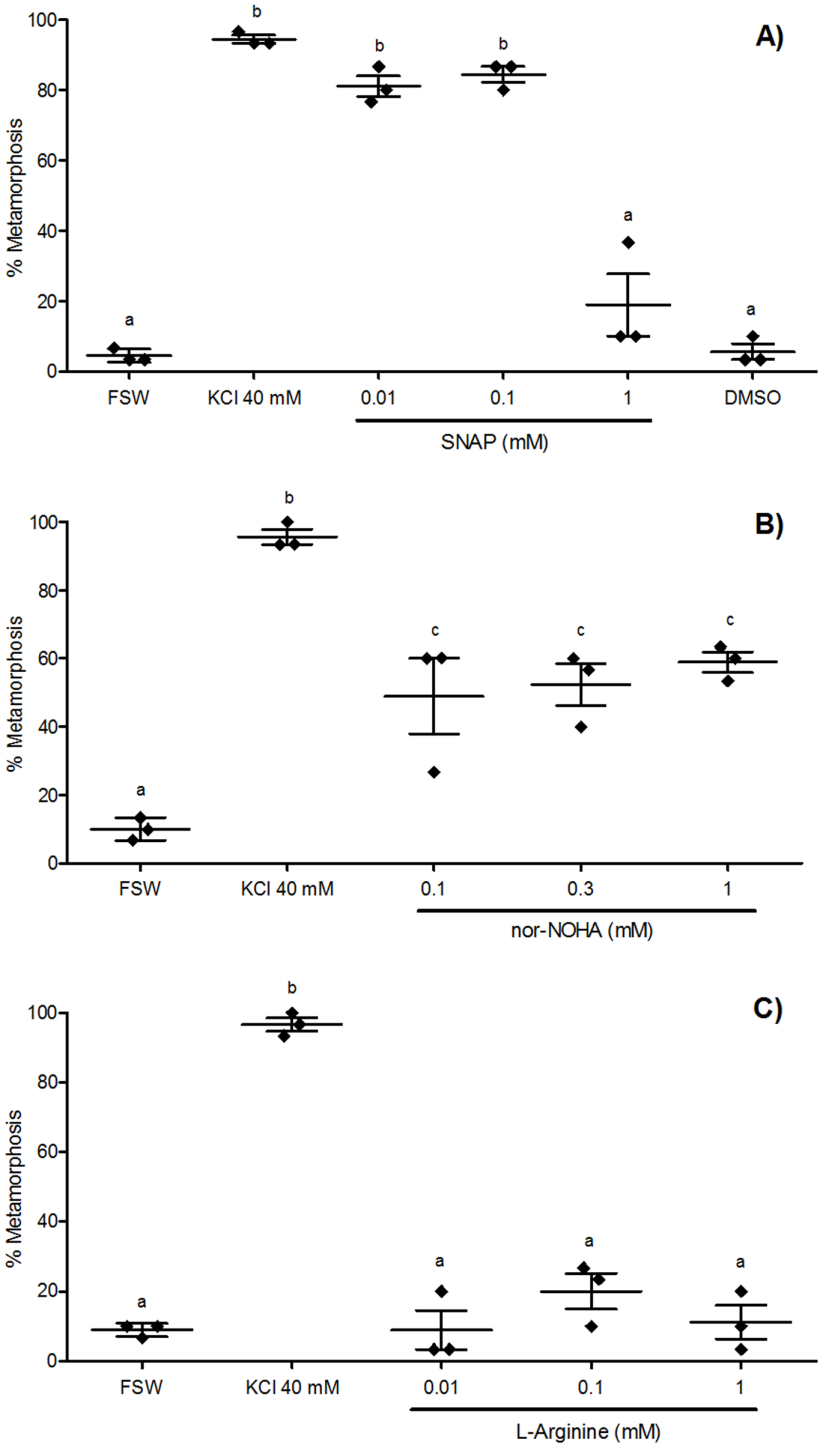
Effect of NO donors on metamorphosis of *Herdmania momus.* (A) S-nitroso-N-acetyl-penicillamine (SNAP), (B) N-hydroxy-nor-arginine (nor-NOHA), and (C) L-Arginine were applied at various concentrations to competent larvae (14 hpf). The number of individuals undergoing metamorphosis was counted 4 h after the initiation of exposure (4 hpi). FSW and 40 mM KCl-elevated FSW were used as negative and positive controls, respectively. Data are presented as the mean percentage of larval metamorphosis ± SEM (n = 3 for each treatment, 30 larvae per replicate). Diamonds indicate the actual percentages of larval metamorphosis in each replicate. Letters above error bars indicate statistically significant differences (P<0.05), as determined by one-way analysis of variance and Tukey’s HSD *post hoc* testing.

An NO donor, SNAP, induced a significantly higher mean percentage of larval metamorphosis compared with FSW at both 0.01 and 0.1 mM (Fig. 3A). In fact, the mean percentages of larval metamorphosis in these concentrations were as high as those observed in the KCl 40 mM positive control (Fig. 3A). There were no significant effects on percent metamorphosis in treatments using 1 mM SNAP, or a solvent control, DMSO (Fig. 3A). A second NO donor, nor-NOHA, significantly induced metamorphosis at all concentrations examined compared with FSW (Fig. 3B). In contrast to SNAP and nor-NOHA, L-arginine was an ineffective NO donor, with no significant effects observable in any of the concentrations examined compared with FSW (Fig. 3C).

### Heat-shock alone is Sufficient to Induce *H. momus* Metamorphosis

**Figure 4:**
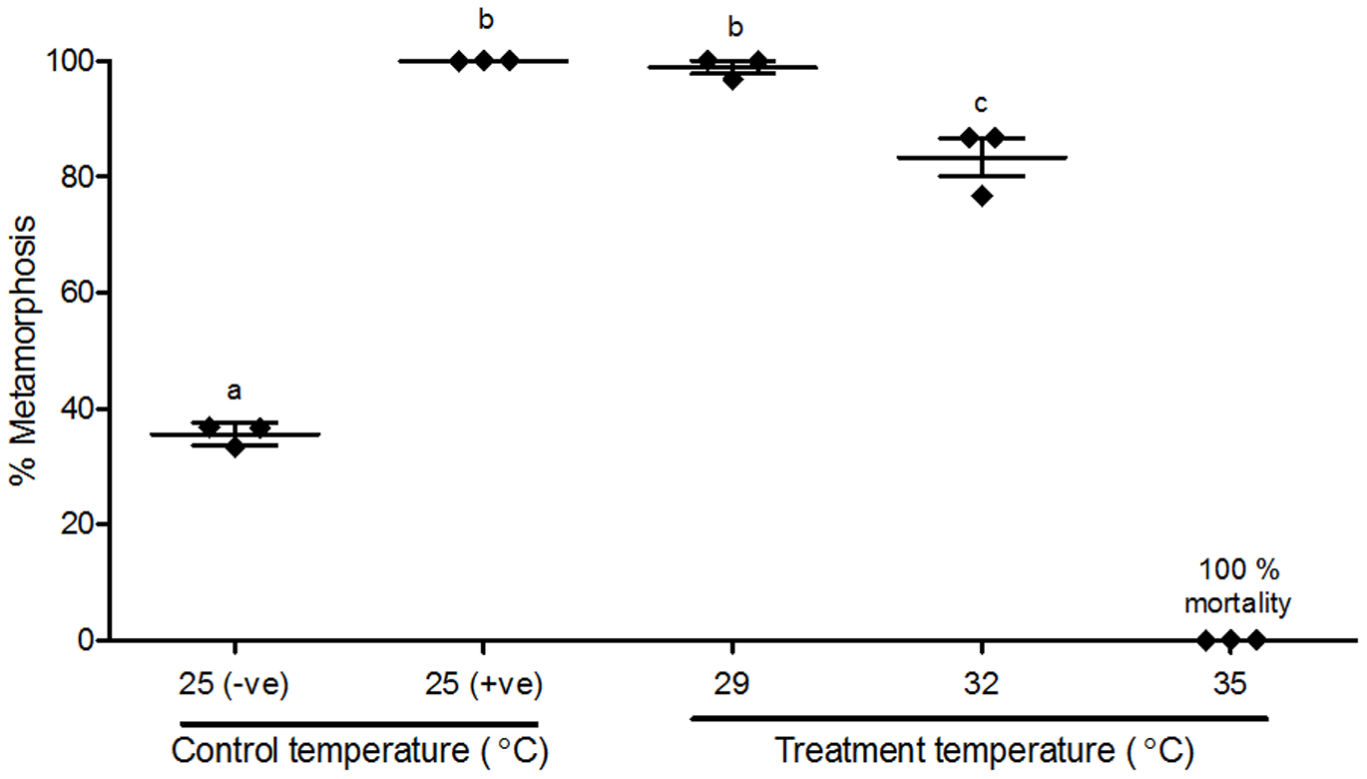
Effect of heat-shock on metamorphosis of *Herdmania momus.* Larvae were exposed at competency (14 hpf) to one of four temperatures (25, 29, 32 and 35°C) for 2 h. The number of individuals undergoing metamorphosis was counted 24 h after the initiation of heat-shock exposure (24 hpi). FSW and 40 mM KCl-elevated FSW, maintained at the culture temperature of 25°C, were used as negative and positive controls, respectively. Data are presented as the mean percentage of larval metamorphosis ± SEM (n = 3 for each treatment, 30 larvae per replicate). Diamonds indicate the actual percentages of larval metamorphosis in each replicate. Letters above error bars indicate statistically significant differences (P<0.05), as determined by one-way analysis of variance and Tukey’s HSD *post hoc* testing.

A heat-shock applied to competent larvae (14 hpf; Fig. 1) for 2 h at temperatures of 29°C and 32°C induced a significantly higher mean percentage of larvae metamorphosing by 24 hpi compared with the 25°C control temperature (Fig. 4). Somewhat surprisingly, the mean percentage of larvae that metamorphosed at 29°C was significantly higher than that at 32°C and, in fact, was as high as the 40 mM KCl positive control (Fig. 4). In the 35°C treatment, analogous to the 10°C temperature elevation that has been shown to induce metamorphosis in other marine invertebrates [7]–[9], no larvae showed any sign of undergoing metamorphosis. Indeed, by 24 hpi, larvae in the 35°C treatment showed no response to gentle stimulation by a needle tip, and showed no metamorphic response even when exposed to the highly inductive 40 mM KCl. Disintegration of larval epidermal tissue was observed shortly after 24 hpi; therefore, larvae in the 35°C treatment were regarded as suffering 100% mortality by 24 hpi (Fig. 4).

**Figure 5:**
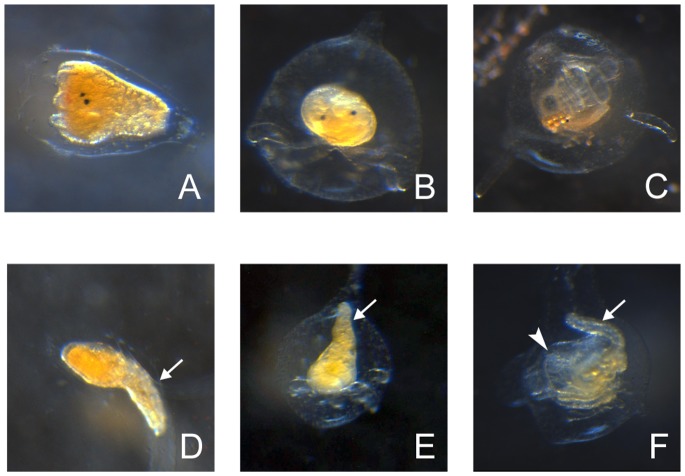
Normal (25°C) and abnormal (32°C) post-larval development of *Herdmania momus.* (A) Normal 4 hpi post-larva. Black eyespots and ampullae growth are visible at the anterior end, left. (B) Normal 24 hpi post-larva. Ampullae are fully extended, bottom. (C) Normal 84 hpi post-larva. Adult morphology has been formed by this time; branchial basket and siphons can clearly be seen. (D) Abnormal 4 hpi post-larva. Arrow indicates incompletely resorbed tail at posterior end. (E) Abnormal 24 hpi post-larva. Arrow indicates some larval tail still remaining. (F) Abnormal 84 hpi post-larva. Arrow indicates some larval tail still remaining; arrowhead indicates deformed branchial basket. Scale bars: A-F, 100 µm.

Interestingly, several individuals that initiated metamorphosis in the 29°C and 32°C treatments showed abnormal morphologies (Fig. 5D–F) compared with those metamorphosing at 25°C (Fig. 5A–C). The abnormal morphologies tended to appear more in 32°C treatment than in 29°C treatment, and typically included incomplete tail resorption and deformed branchial basket (Fig. 5D–F).

### *HmNOS* and *HmHSP90* Expression Implicates Involvement of both Genes at the Initiation of Metamorphosis

To assay the expression profiles of *NOS* and *HSP90* in *Herdmania momus*, we first had to isolate partial genes using degenerate PCR, and determined gene sequences against which we could design specific primers for quantitative reverse-transcriptase PCR (qRT-PCR). This was achieved successfully for both genes of interest.

Degenerate PCR based on animal NOS gene sequences in the NCBI GenBank database yielded a total of 386 bp of *H. momus* sequence that was confirmed by tBLASTx to represent a partial animal NOS gene. This sequence has been named as *HmNOS* and submitted to NCBI GenBank (KC571822); it has very high BLAST similarity to chordate NOS genes, including the ascidian *Ciona intestinalis* neuronal NOS (nNOS) (XM002120231, E-value: 3e-32). The NCBI Conserved Domain Database (CDD) confirmed that *HmNOS* contains an FMN binding domain, which is a highly conserved functional domain of NOS (Fig. S1). Degenerate PCR based on animal HSP90 gene sequences in the NCBI GenBank database yielded a total of 540 bp of sequence that was confirmed by tBLASTx to represent a partial animal HSP90 gene. This sequence has been named as *HmHSP90* and submitted to NCBI GenBank (KC571823); it has very high BLAST similarity to the HSP90 gene of the ascidian *Microcosmus squamiger* (FN984760, E-value: 1e-99). The translated AA sequence of *HmHSP90* contains three highly conserved HSP90 family signature sequences [48] (Fig. S2).

**Figure 6:**
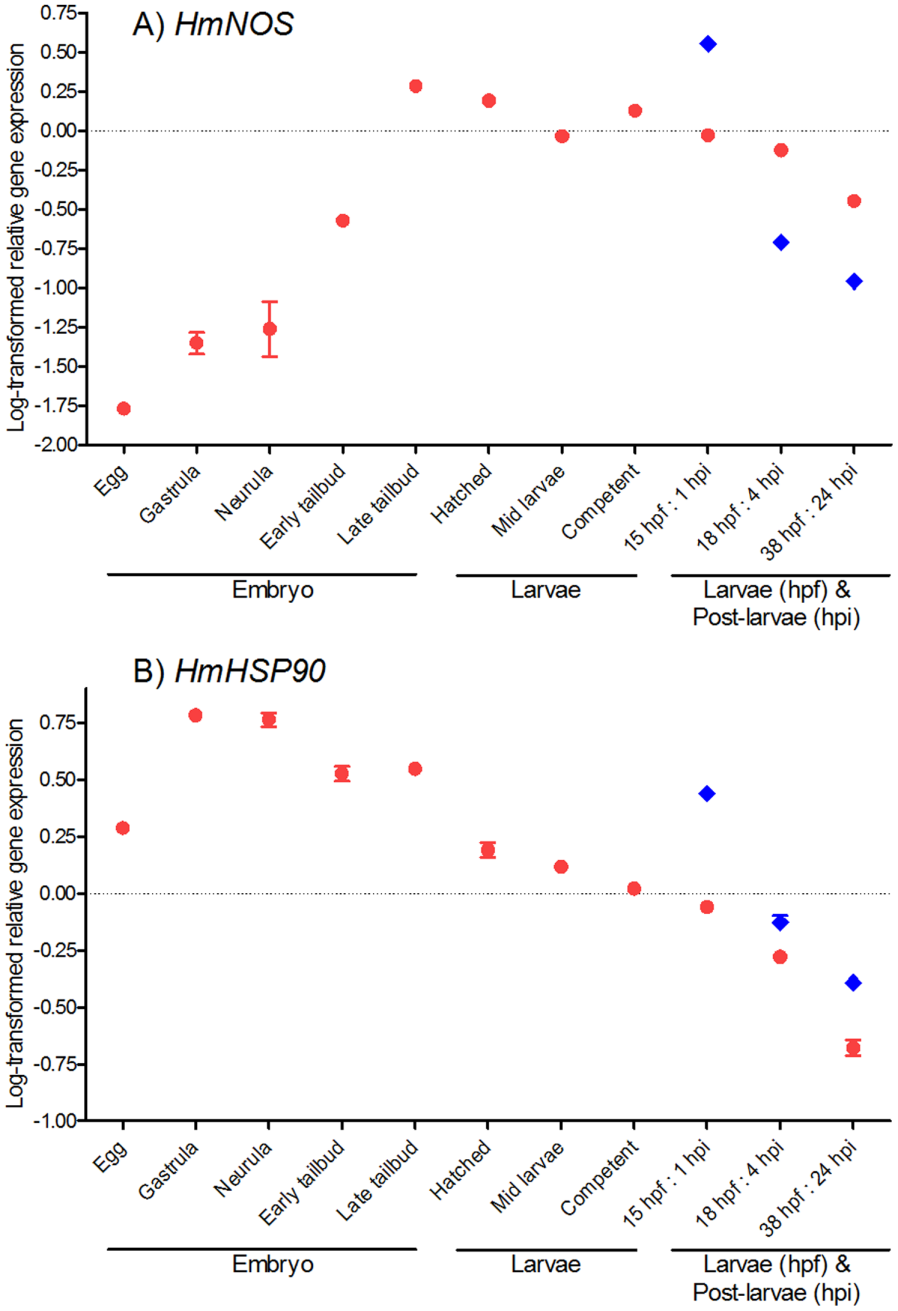
*HmNOS* and *HmHSP90* gene expression through *Herdmania momus* normal development. Transcript abundance was assessed by qRT-PCR using mRNA purified from a pool of ∼200 embryos or larvae for each developmental stage (red circles). Transcript abundance in pooled samples of spontaneously metamorphosed post-larvae is denoted by blue diamonds. Data are presented as log-transformed mean ± SEM of three technical replicates.

The expression of both *HmNOS* and *HmHSP90* during embryonic, larval, and post-larval development (Fig. 1) was measured by qRT-PCR (Fig. 6). Both genes are expressed in all developmental stages examined. During embryonic development, the expression of *HmNOS* increases to a maximum in late tailbud embryos, which is shortly before larval hatching (Fig. 6A). Subsequently in larval samples, *HmNOS* expression generally decreases gradually, except for a slight increase at competency (Fig. 6A). In post-larval samples (that have initiated metamorphosis), *HmNOS* expression increases sharply within the first hour of initiating metamorphosis (as assessed by commencement of tail resorption) (1 hpi; Fig. 6A) but then decreases as metamorphosis progresses, to levels below that observed in non-metamorphosing larvae of the same developmental age (Fig. 6A).

For *HmHSP90*, transcript abundance is relatively high in the egg and increases steeply to a maximum in gastrulas and neurulas (Fig. 6B). After neurulation, *HmHSP90* expression steadily decreases through the remainder of embryonic and larval development, except for a slight increase at late tailbud stage (Fig. 6B). In post-larval samples (that have initiated metamorphosis), the *HmHSP90* expression profile is similar to that of *HmNOS*; that is, *HmHSP90* expression increases sharply within the first hour (1 hpi; Fig. 6B) but then decreases as metamorphosis progresses. In contrast to *HmNOS*, however, *HmHSP90* expression levels in post-larvae remain above those observed in non-metamorphosing larvae of the same developmental age (Fig. 6B).

### Pharmacological Agents Alter *HmNOS* and *HmHSP90* Gene Expression

**Figure 7:**
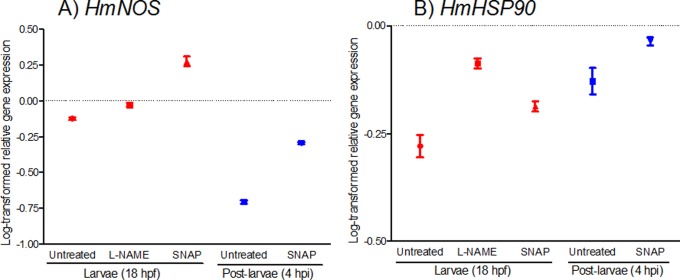
Effect of NO-disrupting pharmacological agents on *HmNOS* and *HmHSP90* gene expression. Competent larvae (14 hpf) were exposed to either 10 mM L-NAME (NOS inhibitor) or 0.1 mM SNAP (NO donor), or were not exposed (untreated control), and sampled 4 hours later as either larvae (18 hpf; had not initiated metamorphosis) or post-larvae (4 hpi; had initiated metamorphosis). All samples depicted here are thus of the same developmental age. Transcript abundance was assessed by qRT-PCR using mRNA purified from a pool of larvae (red circles) or post-larvae (blue diamonds). Data are presented as log-transformed mean ± SEM of three technical replicates.

To assess if pharmacological agents affect normal developmental gene expression, we analysed *HmNOS* and *HmHSP90* expression at 4 h after exposure to either 10 mM L-NAME or 0.1 mM SNAP (Fig. 7); these concentrations were chosen because they gave the greatest effects in the settlement assays (Figs. 2, 3). Indeed, 10 mM L-NAME suppressed metamorphosis of >95% of larvae (Fig. 2A), so that we could only assay gene expression in non-metamorphosed larvae in this treatment. For 0.1 mM SNAP exposure (Fig. 3A), both larval and post-larval samples were collected for gene expression assays.

Exposure of larvae to the NOS inhibitor L-NAME increases *HmNOS* expression compared with non-exposed larvae of an equivalent developmental age (18 hpf; Fig. 7A). Exposure to the NO donor SNAP also causes increased *HmNOS* expression in larval and post-larval samples compared with non-exposed samples of the same developmental age (Fig. 7A). Non-exposed larvae at 18 hpf show higher *HmNOS* expression than non-exposed post-larvae at 4 hpi, and this relationship is retained in SNAP-exposed samples, although the overall magnitude of expression is elevated (Fig. 7A).

Expression of *HmHSP90* is also affected by both L-NAME and SNAP exposures (Fig. 7B). Exposure of larvae to L-NAME increases *HmHSP90* expression compared with non-exposed larvae of an equivalent development age (18 hpf; Fig. 7B). Exposure to SNAP also increases *HmHSP90* expression in both larval and post-larval samples compared with larvae at 18 hpf and post-larvae at 4 hpi, respectively (Fig. 7B). Non-exposed larvae at 18 hpf show lower *HmHSP90* expression than non-exposed post-larvae at 4 hpi, and this relationship is retained in SNAP-exposed samples, although the overall magnitude of expression is elevated (Fig. 7B).

### Heat-shock Alters *HmNOS* and *HmHSP90* Gene Expression

*Herdmania momus* larvae heat-shocked at 29, 32 and 35°C were analysed by qRT-PCR to reveal the effects of heat-shock on *HmNOS* and *HmHSP90* expression during metamorphosis. Timing of RNA sampling for this assay is described in Figure 1.

**Figure 8:**
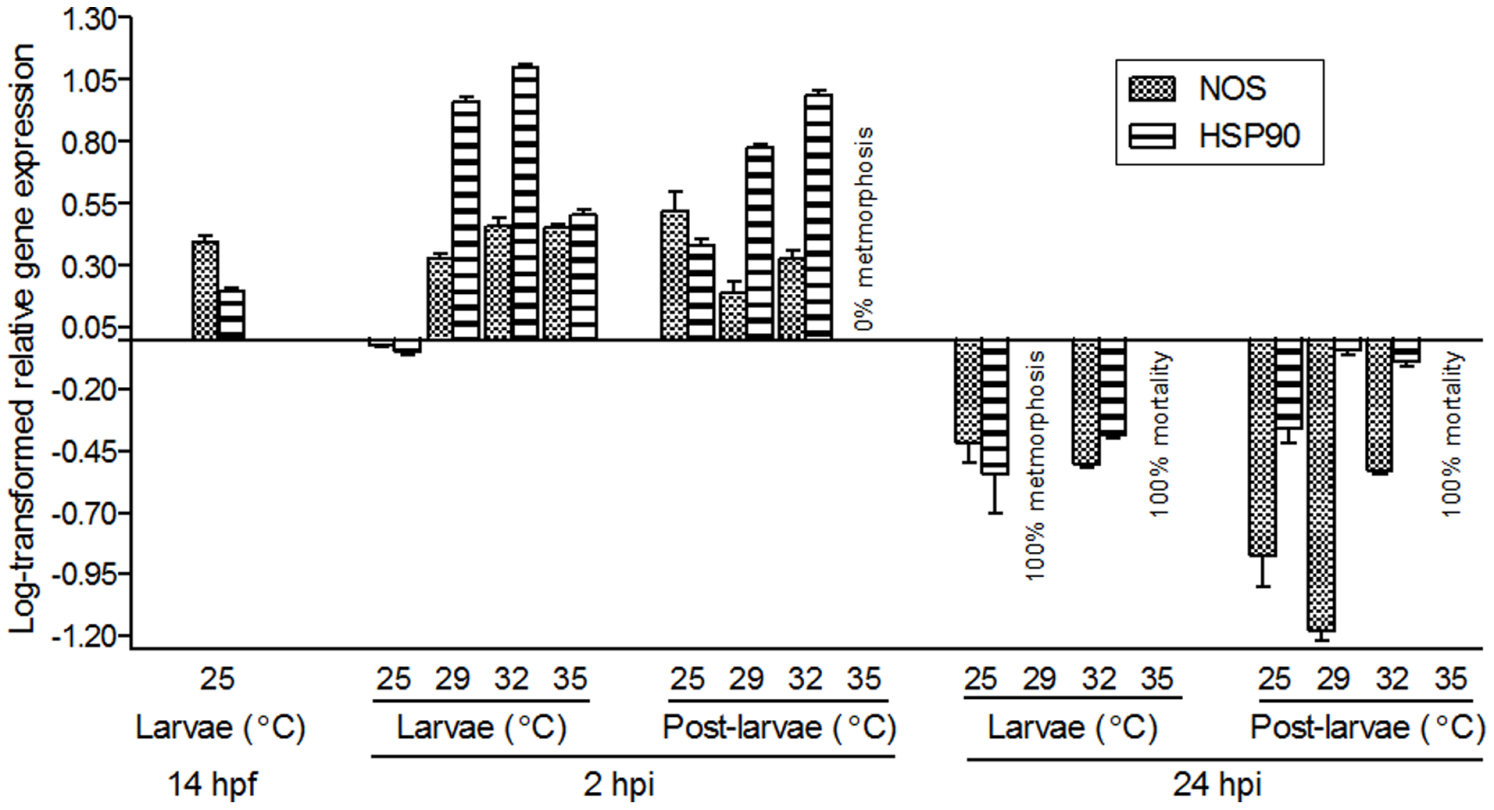
Effect of heat-shock on *HmNOS* and *HmHSP90* gene expression. Competent larvae (14 hpf) were exposed to one of four heat-shock temperatures (29, 32 and 35°C) or retained at the control temperature (25°C) for 2 h. Samples for RNA were collected at the start of the experiment as larvae (14 hpf), at 2 h after the initiation of the heat shock (2 hpi) as either larvae (had not initiated metamorphosis) or post-larvae (had initiated metamorphosis), and at 24 h after the initiation of the heat shock (24 hpi) as either larvae (had not initiated metamorphosis) or post-larvae (had initiated metamorphosis). Transcript abundance was assessed by qRT-PCR using mRNA purified from pools of larvae or post-larvae. Data are presented as log-transformed mean ± SEM of three technical replicates.

In larvae at 2 h post induction (hpi) by heat-shock, *HmNOS* expression in all temperature treatments is higher than in the 25°C control temperature treatment (Fig. 8); expression in the 29°C treatment is slightly lower than in other temperature treatments, but still substantially higher than the 25°C control (Fig. 8). Expression levels in 32 and 35°C treatments are similar to each other (Fig. 8). In post-larvae at 2 hpi, *HmNOS* expression is now lower in the 29 and 32°C treatments than in the control temperature, largely because *HmNOS* expression has been up-regulated in the 25°C control post-larvae compared to non-metamorphosing larvae of the same developmental age (Fig. 8). Here again, expression of *HmNOS* in the 29°C treatment is lower than in the 32°C treatment (Fig. 8). We did not observe any individuals undergoing metamorphosis in 35°C treatment so that no post-larval sample could be collected. By 24 hpi, *HmNOS* expression in non-metamorphosing larvae in the 32°C treatment is lower than that in the control temperature (Fig. 8); all individuals in 29°C treatment were undergoing metamorphosis, so that only post-larvae could be sampled. In these post-larvae at 24 hpi, *HmNOS* expression in the 29°C treatment is lower than in control temperature; however, expression in the 32°C treatment is higher than in the control temperature (Fig. 8). As described above, 100% mortality was observed in the 35°C treatment at 24 hpi.

Expression of *HmHSP90* in larvae at 2 hpi in all temperature treatments show higher levels than the control temperature (Fig. 8). Expression increases in a temperature-dependent manner among control, 29 and 32°C treatments; however, expression in the 35°C treatment is lower than that in the 29 and 32°C treatments (Fig. 8). In post-larval samples at 2 hpi, *HmHSP90* expression increases in a temperature-dependent manner, except for the 35°C treatment (Fig. 8). In larval samples at 24 hpi, *HmHSP90* expression in the 32°C treatment is higher than that in the control temperature (Fig. 8). Expression of *HmHSP90* in both the 29 and 32°C treatments is higher than that in the control temperature in post-larval samples at 24 hpi (Fig. 8).

## Discussion

### In Contrast to other Ascidians, Nitric Oxide Induces Metamorphosis in *Herdmania momus*

Past studies have reported a negative (repressive) regulatory role for NO in early metamorphosis (tail resorption) of ascidian species *Boltenia villosa*, *Cnemidocarpa finmarkiensis*, and *Ciona intestinalis* [38], [39]. In stark contrast to these previous studies, our metamorphosis assays with pharmacological agents revealed a positive (inductive) regulatory role of NO in settlement and metamorphosis of the tropical solitary ascidian *Herdmania momus*. Specifically, the application of all three NOS inhibitors, L-NAME, AGH and SMIS, led to rates of metamorphosis that were either similar to or significantly less than rates of spontaneous metamorphosis in the untreated control. L-NAME significantly reduced spontaneous metamorphosis in a concentration-dependent manner, while AGH and SMIS had no significant effect on spontaneous metamorphosis (Fig. 2). It is worth noting that the effect of AGH and SMIS on metamorphosis was only assessed in relation in cohorts of larvae undergoing spontaneous metamorphosis in FSW. As this varies between cohorts and ranged from about 5–30%, inhibition needs to be pronounced. For example, although less than 2% of the larvae underwent spontaneous metamorphosis in the presence of 0.05 mM SMIS, the rate of spontaneous metamorphosis was only about 6% and thereby there was no significant difference between treatment and control.

Consistent with this effect of NOS inhibitors, the application of NO donors had the effect of inducing metamorphosis, providing compelling support for the action of NO as a positive regulator of metamorphosis in this species. One NO donor, SNAP, was an effective metamorphic inducer at lower concentrations only, suggesting a negative impact of excess NO on the initiation of *H. momus* metamorphosis (Fig. 3A). Another NO donor, nor-NOHA, was an effective metamorphic inducer at all three concentrations tested, with no significant differences in mean percent metamorphosis among all concentrations (Fig. 3B). Nor-NOHA is an arginase inhibitor; therefore, it increases the bioavailability of endogenous arginine, which is an essential substrate of NOS and is catalysed to L-citrulline and NO [21], [49]. Continued inhibition by nor-NOHA of arginase activity can thus result in excess NO production, to the point where physiological processes will trigger a cessation of NOS activity prior to it reaching toxic levels. On the other hand, SNAP externally donates NO itself; therefore, high concentration of SNAP can cause excess (potentially toxic) availability of NO. Thus the highest concentration (1 mM) produces different results in each of these NO donors. Exposure to SNAP at 1 mM induced only 18.89±8.89% larval metamorphosis (not significantly different from spontaneous rates of metamorphosis), but exposure to 1 mM nor-NOHA induced significantly higher 58.89±2.94% metamorphosis (Fig. 3A, B). Lower mean percentages of larvae metamorphosed in nor-NOHA treatments compared with SNAP treatments at the lower concentrations (0.01 and 0.1 mM) can also be explained by an indirect increase of endogenous NO via inhibition of arginase activity by nor-NOHA. Similar concentration-dependent sensitivities to NO have been reported in other aquatic invertebrates. In the pond snail *Lymnaea stagnalis*, the rate of embryonic development varies with NO concentration [50]. While 0.1 mM SNAP and sodium nitroprusside (both NO donors) enhanced embryonic development of *L. stagnalis*, 1 mM treatments proved toxic [50]. Some concentrations of the NO inhibitor L-NAME have negative effects on *L. stagnalis* [50], the sea urchin *Strongylocentrotus purpuratus* [57] and murine [58] embryonic development.

Interestingly in the context of our results, Ercolesi *et al*. [51] recently reported that the NOS inhibitor 1-(2-trifluoromethylphenyl) imidazole (TRIM), when applied to pre-competent larvae, delays later stages of metamorphosis in *C. intestinalis*. The same study also showed that the slow releasing NO donor (Z)-1-{N-[3-Aminopropyl]-N-[4-(3-aminopropylammonio) butyl]-amino}-diazen-1-ium-1,2-diolate (spermine NONOate, SPER/NO), when applied to pre-competent larvae, increases rates of later stages of metamorphosis. Both observations are more consistent with our own observations on the role of NO as an inducer of initiation of metamorphosis in *H. momus*. However, the different temporal focus of the two studies makes explicit comparison difficult beyond this general statement.

### *NOS* and *HSP90* Expression Profiles in *Herdmania momus* are Consistent with an Activating Role in Metamorphosis

Developmental gene expression profiles of *HmNOS* and *HmHSP90* are consistent with the involvement of NO in the initiation of *H. momus* metamorphosis. *HmNOS* transcript levels increase through embryonic development and then effectively plateau from just prior to larval hatching through larval development, while *HmHSP90* transcript levels peek during neurulation and then decreases as development progresses; it is unknown how protein and mRNA levels are related in these two genes. Importantly, both *HmNOS* and *HmHSP90* are up-regulated 1 h after the initiation of metamorphosis compared to equivalent aged swimming larvae (15 hpf; Fig. 6A, B). This supports the hypothesis that both gene products, and thus NO itself, are required for metamorphosis to be successfully initiated, and indeed that NOS and HSP90 interact to induce metamorphosis in *H. momus*. Our data also are consistent with the hypothesis presented in [38] that HSP90 chaperoning of NOS is required for completion of metamorphosis, in that we observe slightly higher levels of *HSP90* expression in metamorphosing post-larvae compared to non-metamorphosing larvae of the same developmental age (Fig. 6B).

No comparable gene expression data are available from other marine invertebrates in which NO acts as a positive regulator of metamorphosis. However, of the species in which NO has been identified as a negative regulator of metamorphosis, NOS expression data is available for some. In contrast to *H. momus*, *NOS* expression decreases from a maximum in the egg (maternal transcripts) to moderate in middle larval stage in the ascidian *C. intestinalis* [39]. This is followed by a sharp up-regulation in late larvae, just prior to initiation of metamorphosis [39]. Because NO has a repressive effect in *C. intestinalis*, the high *NOS* expression could function to maintain the larval state until such time as it is appropriate for metamorphosis to be triggered [39]. Once metamorphosis is underway, *NOS* transcript levels drop dramatically [39], as would be expected in a species where NO acts to represses this transition. This down-regulation of *NOS* immediately after the initiation of metamorphosis is also observed in the mud snail *Ilyanassa obsoleta* [36], again consistent with the idea that NO production needs to be repressed in order for metamorphosis to proceed. In contrast, in the barnacle *Balanus amphitrite*, *NOS* expression is reported as lowest in the cyprid larval stage, just prior to initiation of settlement and metamorphosis [43]. In the slipper shell snail *Crepidula fornicata*, *NOS* expression increases steadily through development to competency at 11 days post release, and then is up-regulated further to a maximum within 6 h post induction of metamorphosis, before decreasing again [52]. Because NO acts as a negative regulator in this species [40], increased *NOS* expression during metamorphosis was interpreted by the authors as a consequence of handling stress during the experiment [52]. These highly variable transcriptional activities of NOS around the time of initiation of metamorphosis are difficult to interpret, not least because of enormous inter-species differences in timing of developmental events and in RNA sampling for gene expression analyses. Our own data reveal just how transient a particular *NOS* expression state may be (see for example the difference between 1 hpi and 4 hpi in Fig. 6A), making it very difficult to meaningfully compare between studies in the absence of very consistent sampling regimes.

### Nitric Oxide Inhibitors and Donors Affect *NOS* and *HSP90* Gene Expression at Metamorphosis

Exposing competent larvae at 14 hpf to the NOS inhibitor L-NAME (10 mM) resulted in increased expression of *HmNOS* and especially of *HmHSP90* in 18 hpf larvae, compared to non-exposed larvae (Fig. 7). Post-larval expression could not be assessed in this experiment because so few larvae initiated metamorphosis under the repressive effect of the 10 mM L-NAME (Fig. 2A). The mechanism underlying the up-regulation of *NOS* in the presence of L-NAME is unclear. Studies in mammalian cells have reported that reducing NO availability by NOS-inhibiting or NO-scavenging agents causes an up-regulation of endothelial or inducible NOS gene expression, suggesting a feedback regulation of NOS transcriptional activity according to endogenous NO levels [53]–[55]. We hypothesise that the application of the NOS inhibitor L-NAME in our study caused a similar compensatory feedback response, resulting in the up-regulation of *HmNOS* expression. Nonetheless, the observed increase in *HmNOS* transcripts did not result in an increased rate of metamorphosis, suggesting that 10 mM L-NAME is sufficient to inhibit both existing NOS and any newly-translated NOS synthesised as a result of the up-regulated *HmNOS* in this treatment.

Both *HmNOS* and (less so) *HmHSP90* are up-regulated in response to SNAP (NO donor) exposure in both the minority of larvae that did not initiate metamorphosis and majority of post-larvae that initiated metamorphosis (not observed for L-NAME treatments). The similar changes in larval and post-larval gene expression profiles suggest that the transcriptional regulation of these genes is sensitive to NO concentration. The up-regulation of *HmNOS* expression in response to an NO donor (SNAP) was unexpected because the same response is seen in response to a NOS inhibitor (L-NAME). In mammalian cells, an increased level of NO by the application of an NO donor triggered a negative feedback response to NOS transcriptional activity, leading to the down-regulation of eNOS or iNOS gene expression [53]–[56]. In our study, the down-regulation of *HmNOS* expression in SNAP exposure might thus be a more reasonable expectation. However, it is also known that application of an NO donor can destabilise mRNA transcription, depending on the donor concentration and duration of exposure [57], [58]. Although the direct effect of NO donors specifically on NOS mRNA destabilisation is not yet understood, it would seem possible that the SNAP concentration used in our experiment could destabilise *HmNOS* mRNAs. This in turn could trigger a compensatory feedback response observed as an up-regulation of *HmNOS* expression. Since the up-regulation of *HmNOS* occurs at the initiation of metamorphosis in normal development (Fig. 6A), further up-regulation of *HmNOS* might be necessary to compensate the destabilised abundance of *HmNOS*. The exact mechanisms of increased *HmNOS* expression in the context of both L-NAME and SNAP exposure remain to be elucidated.

### Heat-shock Induced Metamorphosis Alters *NOS* and *HSP90* Gene Expression Profiles

Heat-shock induction of metamorphosis has been reported previously in *H. momus* and *C. intestinalis*, as well as in molluscs and a cnidarian [6]–[9], [44]. Consistent with previous observations, increase in temperature to 29 and 32°C significantly increases the percent of larvae initiating metamorphosis and post-larvae undergoing normal metamorphosis; at 35°C all post-larvae died by 24 hpi (Fig. 4). Degnan *et al*. [44] previously demonstrated a temperature-dependent induction of metamorphosis in *H. momus* up to 31°C, which was as high as that generated by 40 mM KCl-elevated FSW [44]. Our data presented here reveal that the 29°C treatment was equally as effective as 40 mM KCl-elevated FSW, but the 32°C treatment was less effective than either of these (Fig. 4). These results indicate that the optimal temperature for heat-shock induction of *H. momus* metamorphosis lies between 29–31°C, and that 35°C is beyond the thermotolerance of competent *H. momus* larvae.

Expression profiles of both *HmNOS* and *HmHSP90* genes during metamorphosis are altered by heat-shock (Fig. 8), suggesting that *H. momus* larvae can modulate their molecular pathways to achieve successful metamorphosis under variable environmental conditions. In 2 hpi larvae (i.e. the minority that did not initiate metamorphosis), expression of *HmHSP90* dramatically increased in 29 and 32°C treatments and less so in the 35°C treatment (all larvae exposed to 35°C died by 24 hpi without showing any initiation of metamorphosis; Figs. 4, 8). Tomanek and Somero [59] reported that the ability to express HSP90 is strongly related to thermotolerance of the turban snail, *Tegula* sp. In this context, down-regulation of *HmHSP90* expression in the 35°C treatment is entirely consistent with our conclusion from the metamorphosis assay that 35°C is beyond the thermotolerance of *H. momus* competent larvae.

Comparison of *HmNOS* and *HmHSP90* transcript levels between 2 hpi larvae and post-larvae reveals that at 25°C both transcripts are significantly more abundant in post-larvae, indicating that both genes being normally up-regulated upon induction of metamorphosis and consistent with NOS having an activating role at metamorphosis (Fig. 8). This is in contrast to the situation at 29 and 32°C, where both *HmNOS* and *HmHSP90* transcript levels are significantly lower in post-larvae compared to larvae. *HmNOS* and *HmHSP90* are up-regulated in heat-shocked larvae. Although this is also the case for *HmHSP90* in post-larvae, *HmNOS* is down-regulated in heat-shocked post-larvae (Fig. 8). Given *HmNOS* transcript abundance normally decreases between 1 and 4 hpi (Fig. 6), the differences between heat-shock and normal transcript levels may reflect temperature-dependent rates of metamorphosis (i.e. 29 and 32°C are developing faster).

However, these gene expression patterns in the 29 and 32°C treatments are inconsistent with the observation that the 29°C heat-shock was significantly more effective at inducing metamorphosis than the 32°C heat-shock, such that we expected to see higher *HmNOS* expression at 29°C than at 32°C. In fact, we saw the opposite. It is known that, under conditions of cellular stress, HSP90 function is diverted from its constitutive chaperon activity (that is, stabilising NOS and other client proteins) to re-folding the denatured proteins that accumulate under cellular stress; this results in an attenuated NOS function. Given that 32°C is getting close to the larval thermotolerance of *H. momus*, the majority of available HSP90 may be involved in repairing the denatured proteins. Therefore, even though *HmNOS* expression at 32°C is higher than that at 29°C, actual NO synthesis may be mitigated by the diverted function of HSP90, resulting in reduced metamorphosis under 32°C compared to 29°C heat-shock.

At 24 hpi, larvae and post-larvae that had been exposed to a 2 h heat-shock had been developing at ambient temperature (25°C) for 22 h. While *HmNOS* and *HmHSP90* expression levels are essentially the same in control (25°C) and 32°C heat-shocked larvae, in 24 hpi post-larvae *HmHSP90* remains higher in 29 and 32°C heat-shock treatments compared to 25°C controls (Fig. 8). *HmNOS* expression in slightly lower in 29°C heat-shock treatments compared to 25°C controls but 32°C levels are significantly higher. At this temperature, abnormal morphologies, including incomplete tail resorption and deformed branchial baskets are observed (Fig. 5). Abnormal morphologies have been observed in other marine invertebrates exposed to similar heat-shock regimes including the slipper shell snail *C. fornicata*, in which individuals exhibited variable morphological changes including to the velar lobes [8]. These results suggest that heat-shock can directly affect developmental pathways during marine invertebrate metamorphosis, resulting in modified phenotypes. Rutherford & Lindquist [60] first reported that attenuated HSP90 chaperone function for developmental signalling proteins by exposure to heat-shock can reveal cryptic genetic variations, which manifest as heritable abnormal morphological traits in *D. melanogaster*. Similar phenomena have since been reported in *Arabidopsis thaliana* and *Danio rerio* [61], [62]. On this basis, we speculate that mitigated constitutive HSP90 chaperone function by heat-shock causes compromised developmental signalling protein activity during *H. momus* metamorphosis, leading to the appearance of abnormal morphologies.

### Could the Regulation of Metamorphosis vary between Ascidian Species?

The unexpected outcome from this study is that of the positive regulatory role of NO in *H. momus* metamorphosis, in direct contrast to the negative regulatory role reported from all other ascidian species so far [38], [39]. Among ascidians, *H. momus* and *C. intestinalis* are the best studied in the context of molecular mechanisms underlying metamorphosis [17], [45], [63]–[70] and the data we present here reveal that the nature of NO regulation in metamorphosis of these two species markedly differs.

In *H. momus*, initiation of metamorphosis is regulated by *Hemps*, which encodes a novel secreted protein containing 4 EGF-like repeats and 3 novel cysteine-rich repeats [71]. In competent larvae, *Hemps* is expressed in the anterior signalling centre. During early metamorphosis, Hemps protein diffuses posteriorly [17]. Application of anti-Hemps antibody impedes the process of metamorphosis [17]. There is no ortholog of *Hemps* in the genome of *C. intestinalis* [72]. Instead, the related C*i-meta1* gene, which encodes a secreted protein with 6 EGF-like repeats and 13 calcium-binding EGF-like repeats has been identified [64]. It is localised to the anterior region of post-larvae immediately after the initiation of metamorphosis [64]. Although this suggests these are similar, microarray profiling of *H. momus* during metamorphosis has revealed that 40% of the genes that are differentially expressed have no significant match with genes in the *C. intestinalis* genome [67]. Together, these data strongly suggest that the underlying transcriptional mechanisms that regulate metamorphosis in *H. momus* and *C. intestinalis* are significantly different from each other [67]. This observation may also reflect the divergent role of NO as a positive or negative metamorphic regulator in these species.

In *C. intestinalis*, tail resorption involves apoptosis, which is regulated by activation of caspase-3-dependent proteins [66]. Chambon *et al*. [69] showed that activation of the extracellular signal-regulated kinase (ERK) and the c-Jun NH2-terminal kinase (JNK) of mitogen activated protein kinase (MAPK) are required for the apoptosis during metamorphosis. Comes *et al*. [39] demonstrated significantly increased caspase-3 enzymatic activity in *C. intestinalis* larvae treated with a NOS inhibitor. They also showed co-localisation of gaseous endogenous NO and activated caspase-3 signals in the tail extremity at the initiation of tail resorption [39]. As tail resorption proceeds, activated caspase-3 signal is detectable throughout the tail, whereas the NO signal becomes almost undetectable [39]. These results suggest that NO regulates apoptotic events during *C. intestinalis* tail resorption via modulation of caspase-3 activity. Nitric oxide is known to inhibit caspase-3 activity by S-nitrosylation redox modification of the catalytic site [73]. This caspase-3 inhibition by NO is found in murine hepatocytes and endothelial cells [74], [75]. In other murine studies, NO also acts as a proapoptotic molecule through several different pathways, including increased ceramide generation, accumulation of p53, and activation of JNK/stress-activated protein kinase (SAPK) of the MAPK pathway, which activates caspase-3 via release of cytochrome c from mitochondria to cytosol [76]–[78]. That is, NO has both pro- and antiapoptotic activities, depending upon specific concentrations of NO, cell types, and redox state [73]. Here, we propose that the regulatory switch that underlies the apparent versatility of NO to either induce or inhibit apoptosis may be similar to that which underlies the dichotomous role of NO in *H. momus* and *C. intestinalis* as a either positive or negative regulator of metamorphosis. The same regulatory mechanisms that determine NO as a proapoptotic molecule in the mammalian systems may have been specifically adopted by *H. momus,* whereas *C. intestinalis* adopted the regulatory system that uses NO as an antiapoptotic molecule to repress the initiation of metamorphosis. The specific involvement of apoptosis in metamorphosis of *H. momus* has not yet been investigated.

## Conclusions

By coupling pharmacological treatments with the analysis of *HmNOS* and *HmHSP90* gene expression, we present compelling evidence of a positive regulatory role for NO in metamorphosis of the solitary tropical ascidian *H. momus*. The outcome of our investigations unexpectedly contrasts with results from other ascidian species, in which NO has been consistently reported as a negative regulator of metamorphosis. The exposure of competent larvae to NOS inhibitors or NO donors results in an up-regulation of *NOS* and *HSP90* genes. Heat-shock of competent larvae induces metamorphosis in a temperature dependent manner, up to a thermal tolerance that approaches 35°C. Both larval/post-larval survival and the appearance of abnormal morphologies in *H. momus* post-larvae reflect the magnitude of up-regulation of the *HSP90* gene in response to heat-shock. The role of NO as a positive metamorphic regulator in *H. momus* suggests the existence of inter-specific adaptations of NO regulation in ascidian metamorphosis.

## Materials and Methods

### *H. momus* Larval Culture

Reproductive adult specimens of *H. momus* were collected from Heron Island Reef, Great Barrier Reef, Australia (23°27′S; 151°55′E), under research permit G12/35053.1 issued by the Great Barrier Reef Marine Park Authority. Detailed protocols for maintenance of the collected specimens, fertilisations, and larval collection and culture in the laboratory were followed in accordance with Degnan *et al*. [44], [79] and Degnan and Johnson [80]. In brief, the collected adult *H. momus* were held in an aquarium with an aerator or flow-through seawater system for a minimum of three days under constant light to allow them to accumulate gametes [79]. Fertilisation was achieved by strip spawning, pooling the gametes from testes and ovaries surgically removed from at least three individuals. Fertilised eggs and larvae were cultured in 0.2 µm filtered seawater (FSW) at 25±0.5 °C until the competent state was reached at 14 h post fertilisation (hpf) [44].

### Metamorphosis Assay with NO-disrupting Pharmacological Agents

Pechenik *et al*. [40] and Biggers *et al*. [42] reported different metamorphic responses to specific NOS inhibitors in the mollusc *Crepidula fornicata* and the annelid *Capitella teleta*. Therefore, we elected to test several different NOS inhibitors and NO donors to ascertain the role of NO in the initiation of *H. momus* metamorphosis (Table 1). As NOS inhibitors, we used L-nitroarginine-methyl-ester (L-NAME) (Sigma), aminoguanidine hemisulfate (AGH) (Sapphire Bioscience), and S-methylisothiourea sulphate (SMIS) (Sapphire Bioscience). We used S-nitroso-N-acetyl-penicillamine (SNAP) (Sapphire Bioscience) as a direct NO donor. L-Arginine (Sigma), which is the substrate of NOS enzymatic reaction, and the arginase inhibitor, N-hydroxy-nor-arginine (nor-NOHA) (Sapphire Bioscience), were also applied as NO donors since they theoretically increase the internal concentration of arginine, leading to increasing availability of NOS substrate [21], [39]. All chemicals used here have been demonstrated to affect the induction of metamorphosis in other ascidians, molluscs, an annelid, an echinoderm, and a crustacean [36]–[43].

Stock solutions of 0.5 M L-NAME, 0.5 M AGH, 0.5 M SMIS, 0.5 M L-Arginine and 0.1 M nor-NOHA were prepared in FSW, stored at 4°C, and diluted to final experimental concentrations just prior to the experiments. For SNAP, a stock solution of 0.1 M was prepared in dimethyl sulfoxide (DMSO) immediately preceding the experiment and diluted to final concentrations just prior to use. The final concentrations of each chemical used in the experiments are listed in Table 1.

Metamorphosis assays were initiated at competency (14 hpf) (Fig. 1) and performed in 6-well 35-mm diameter sterile polycarbonate tissue culture dishes with 10 ml of FSW per well. Experiments with each pharmacological agent contained the following controls and treatments: (1) FSW only (negative control), (2) 40 mM KCl-elevated FSW (positive control; [44]), or (3) FSW containing a pharmacological agent (treatments). Three replicates of each treatment with 30 competent larvae per replicate were examined. The number of larvae that had initiated metamorphosis, as defined by 50% tail resorption or later developmental stages [81] was counted at 4 h post induction (hpi) [44], [67]. Also at this time, non-metamorphosed larvae and metamorphosing post-larvae were transferred into TRI reagent (Sigma) and stored at −80°C for later isolation of total RNA for gene expression assays (see below).

### Metamorphosis Assay with Heat-shock Treatments

For heat-shock assays, we chose 29, 32 and 35°C as the treatment temperatures with the following rationalisations: 29°C was the highest water temperature measured within the depth of 0.3 m during the mid-day low tide on Heron reef flat during the period of larval culture, 32°C was the average of the highest water temperature at 0.3 m depth recorded in 2009 (33.02°C) and 2010 (31.21°C) on Heron Island Reef flat (http://data.aims.gov.au/aimsrtds/datatool.xhtml?site=130), and 35°C represented the 10°C temperature elevation that has been shown to induce metamorphosis in other marine invertebrates [7]–[9].

Heat shock metamorphosis assays were initiated at competency (14 hpf) (Fig. 1) and performed in 6-well 35-mm diameter sterile polycarbonate tissue culture dishes with 10 ml of FSW per well. Three replicates of each treatment with 30 competent larvae per replicate were examined. Three water baths were heated to the experimental temperatures with aquarium heaters. The culture dishes with FSW were floated in the water baths to reach the experimental temperatures prior to the induction of metamorphosis. At 14 hpe, larvae were quickly transferred into the wells by pipetting and were exposed to the experimental temperatures for 2 h. Then, the plates were removed from the water baths and gradually cooled back to 25°C. A negative control (FSW only) and a positive control (40 mM KCl in FSW) maintained constantly at the 25°C culture temperature were also included.

Non-metamorphosed larva and metamorphosing post-larvae were fixed at three different time points during the experiment (Fig. 1) to provide a source of total RNA for gene expressions assays (see below); these time points were 14 hpf at the start of the experiment, 2 hpi to assay initial response, and 24 hpi at the end of the experiment to assay long-term response. Metamorphosis was counted only at the end of the experiment, at 24 hpi, as described above.

### Collection and Preparation of RNA for Quantitative Reverse Transcriptase PCR (qRT-PCR)

To investigate temporal expressional patterns of *NOS* and *HSP90* genes in *H. momus* normal development, total RNA samples were collected during embryonic, larval, and post-larval development (Fig. 1). Embryonic and larval samples were produced as described above under *H. momus* larval culture. These samples are referred as “normal development”. Spontaneously metamorphosing individuals were collected for the normal development post-larval samples, as gene expression in these samples will not have been modified by exposure to any external agents.

To examine how exposure to pharmacological agents during metamorphosis affects underlying *NOS* and *HSP90* gene expression in *H. momus*, we collected larvae and post-larvae at 4 hpi; that is, 4 h after being exposed to either L-NAME or SNAP at concentrations demonstrated in our pharmacological experiments to significantly either inhibited (L-NAME) or induced (SNAP) the mean percentage of *H. momus* larvae metamorphosing.

We also collected RNA samples to test the effects of heat-shock on *NOS* and *HSP90* gene expression during metamorphosis, during the heat-shock experiments described above. For RNA sample collection, 500 ml beakers containing 400 mL FSW were heated to the experimental temperatures prior to the larval transfer. Then, the competent larvae (14 hpf) were transferred into the beakers. The RNA samples were collected at the following time points: at competency (14 hpf, prior to the initiation of heat-shock), at 2 h post induction (at the end of heat-shock; only individuals showing tail resorption were collected), and 24 h post induction (22 h recovery; only individuals showing extended ampullae were collected) (Fig. 1).

For the heat-shock treatments, we collected individual larvae undergoing tail resorption as evidence that metamorphosis had been initiated. These samples could thus include both larvae undergoing spontaneous metamorphosis and larvae undergoing heat shock-induced metamorphosis. To account for the effect of spontaneous metamorphosis in our gene expression analyses, we compared our heat-shock samples against a non-heat shocked (spontaneous metamorphosis only) post-larval sample maintained at the control temperature (25°C). Any differences in gene expression between control samples and heat-shocked samples could be attributed to the effects of the heat-shock.

For all RNA samples, ∼200 individuals were collected for each developmental stage. All the collected samples were preserved in TRI reagent (Sigma) and stored at −80°C prior to extraction. Total RNA from each larval pool was extracted in TRI reagent (Sigma) following the manufacturer’s protocol, and assessed by agarose gel electrophoresis and NanoDrop ND-1000 (Thermo Scientific) UV spectrophotometry. To remove genomic DNA, total RNA (1 µg) was treated with DNase I (Invitrogen) as per the manufacturer’s protocol. Complementary DNA (cDNA) was synthesised from 0.5 µg DNase-treated RNA using Superscript III Reverse Transcriptase (RT) (Invitrogen) following the manufacturer’s protocol. For the assessment of genomic DNA contamination, no-RT control samples were prepared from the 0.5 µg DNase-treated RNA for the all RNA samples and tested by quantitative RT-PCR (qRT-PCR). cDNA and no-RT samples were stored at −20°C.

### Isolation of *HmNOS* and *HmHSP90*

We isolated a single NOS gene from *H. momus* by degenerate PCR. To design the degenerate primers, NOS derived amino acid sequence of the following species were aligned: *Homo sapiens* (AAB49040), *Xenopus laevis* (AAI70183), *Danio rerio* (NP571735), *Branchiostoma floridae* (AAQ02989), *Ciona intestinalis* (XP002120267), *Aplysia californica* (AAK83069), and *Lehmannia valentiana* (BAF73722). The degenerate forward (DegF1) and reverse (DegR1) primers were designed from conserved amino acid (AA) sequences of ILYATETG and VGPGTGIAP, respectively (Fig. S3). The DegF1and DegR1sequences are 5′-ATHYTNTAYGCNACNGARACNGGN-3′ and 5′-NGGNGCDATNCCNGTNCCNGGNCCNAC-3′, respectively. A touch-down PCR profile was used: 94°C for 5 min, 5 cycles at 94°C for 30 sec, 56 to 50°C (2°C increment for every 5 cycles) for 30 sec, and 70°C for 2 min, 25 cycles at 94°C for 30 sec, 48°C for 30 sec, and 70°C for 2 min, and 72°C for 10 min. The obtained PCR product was used as a template to run a nested-PCR. A nested reverse primer (DegR2) was designed from conserved AA sequence of PGDHLGVF, which is located within the amplified region of the DegF1 and DegR1 primer combination (Fig. S3). The primer sequence of DegR2 is 5′-AANACNCCNARRTGRTCNCCNGG-3′. The touch-down PCR cycle was used with a modified parameter at 70°C for 1 min. Each PCR reaction comprised 1×reaction buffer (Promega), 0.2 mM dNTP, 1 U Taq polymerase (New England Biolab), and 1 µM of each primer in a total volume of 20 µL.

We also isolated a single HSP90 gene from *H. momus* by degenerate PCR. To design the degenerate primers, NOS derived amino acid sequence of the following species were aligned: *Homo sapiens* (NP005339), *Bos taurus* (Q76LV2.3), *Danio rerio* (NP571403), *Paralichthys olivaceus* (ABG56393), *Branchiostoma floridae* (XP002249024), and *Crassostrea gigas* (ABS18268). The degenerate forward (DegF) and reverse (DegR) primers were designed from conserved AA sequences of TFAFQA and QFIGYPI, respectively (Fig. S4). The DegF and DegR sequences are 5′-GARCANTTYGCNTTYCARGCNGA-3′ and 5′-ATNGGRTANCCDATRAAYTG-3′, respectively. A touch-down PCR profile was used: 94°C for 5 min, 5 cycles at 94°C for 30 sec, 48 to 42°C (2°C increment for every 5 cycles) for 30 sec, and 70°C for 30 sec, 25 cycles at 94°C for 30 sec, 40°C for 30 sec, and 70°C for 30 sec, and 72°C for 10 min. The mixture of PCR reaction in total 25 µl comprised the final concentrations of 1×reaction buffer (Promega), 2 mM MgCl_2_, 0.2 mM dNTP, 0.5 U Taq polymerase (New England Biolab), and 1 µM of each primer.

Degenerate PCR products were separated and visualised by 1.5% agarose TAE gel electrophoresis. Products of expected size (approximately 800 bp) were excised and gel-purified by a silica suspension method [82]. The purified PCR products were cloned using the pGEM-T Easy Vector System 1 (Promega). Successfully transformed recombinant colonies were picked and directly added to a second PCR mixture with a final concentration of 1×reaction buffer (Promega), 0.5 mM MgCl_2_, 0.2 mM dNTP, 1.5 U Taq polymerase (New England Biolab), and 0.25 µM of both M13 forward and reverse primers to amplify inserts_._ This second PCR reaction used a profile of 94°C for 5 min, 35 cycles of 94°C for 30 sec, 55°C for 1 min, and 72°C for 1 min, with a final extension of 72°C for 10 min. Products were then separated by 1.5% agarose TAE gel electrophoresis and those of correct size were purified using a silica suspension method [82]. Selected clones were Sanger-sequenced using reactions containing 1 µL of Big Dye Terminator mix v 3.1(Applied Biosystems), 1×Big Dye Terminator reaction buffer, 3.2 pmol of primer, and 6–10 ng in a final volume of 10 µL, as recommended by the Australian Genome Research Facility (AGRF). Sequencing reactions were purified by magnesium sulphate precipitation in accordance with the AGRF protocol and submitted to the AGRF Brisbane node (Queensland, Australia). The resulting sequences were compared to orthologs in the National Center for Biotechnology Information (NCBI) by tBLASTx queries.

### qRT-PCR to Assay *HmNOS* and *HmHSP90* Temporal Expression

To analyse the transcriptional profiles of *HmNOS* and *HmHSP90* genes during *H. momus* development (embryonic, larval, and post-larval stages), in response to pharmacological agents and in response to heat-shock (Fig. 1), we performed qRT-PCR using ∼3.75 ng cDNA template in a 15 µL reaction mix that comprised cDNA, SYBR Green Master mix (Roche), and 0.17–0.34 µM primer on a Light Cycler 480 (Roche). The following gene-specific primers were designed to meet criteria of >45% GC content and >60°C primer melting temperature, and to amplify a fragment of 125–220 base pairs (bp): *HmNOS* forward 5′-CGGCAAACTTGAGACTGGC-3′ and reverse 5′-GCGTTTCAACAGTATGCATGC-3′ and *HmHSP90* forward 5′-GTGTCAGCTACCAAGAAGGC-3′ and reverse 5′-AGACATGATCAACAACTTGGG-3′.

To normalise the level of transcription for obtaining relative gene expression values, we used the geometric mean of two reference genes - *Hm-Enonyl Co-A Hydratase* (*Hm-CoA*) (forward 5′-TAGGGGGCTTCAATCGCAA-3′ and reverse 5′-TCCTCCACATCCAACAACC-3′) and *Hm-ubiquitin* (*Hm-Ubiq*) (forward 5′-CCTTGACAGGAAAGACCATCAC-3′ and reverse 5′-GGTTCGATGACGCCACC-3′). These reference genes were previously demonstrated by Hinman and Degnan [83] and Woods *et al*. [67] to maintain uniform transcription throughout *H. momus* development. Their stability across all experimental sample sets was confirmed using Genorm software [84].

The following qRT-PCR reaction parameters were used: initial denaturation 95°C for 10 min (ramp rate 4.4°C/sec), and 40–50 cycles of 95°C for 5 sec (ramp rate 4.4°C/sec), 58°C for 10 sec (ramp rate 2.2°C/sec), and 72°C for 20 sec (ramp rate 4.4°C/sec). Melt curve data acquisition was from 55–95°C with continuous measurement (acquisition/°C = 5), and purity of PCR products was confirmed by the presence of a single peak in the temperature melt curves. All samples were run in triplicate to account for technical variation. For each primer pair, a standard curve was generated to calculate the efficiency of qRT-PCR using a dilution series from the calibrator sample, which was a mixture of 4 µl of all undiluted cDNA samples used in a particular experiment. In addition to the developmental stage cDNAs, a no-template (H_2_O) control and the calibrator sample were included for each qRT-PCR run and for each primer pair. The efficiencies of each primer pair and the cycle threshold of each sample were calculated by the second derivative method using Roche Light Cycler 480 software. Relative expression ratios were calculated as the ratio of gene expression between the gene of interest and the geometric mean of the two reference genes (normalisation factor) relative to their calibrator sample. The expression ratios and standard errors were calculated using REST-RG beta software version 3 [85].

### Statistical Analysis

Data collected from metamorphosis assays were analysed by one-way analysis of variance (ANOVA) with treatment as a factor. Significant differences among treatments were detected by Tukey’s HSD *post hoc* testing. Prior to ANOVA, all data were arcsine-transformed to improve the normal distribution of samples. Levene’s test was performed to ensure homogeneity of variance among treatments. All statistical analyses were performed in R (R Foundation for Statistical Computing). An alpha value of 0.05 was used to determine a significant difference [86].

## Supporting Information

Figure S1 **Multiple sequence alignment of translated amino acid sequence of HmNOS with other animals.** Black shading indicates completely conserved residues; grey indicates partially conserved residues. FMN delineates the conserved flavin mononucleotide domain. Hm *Herdmania momus*, Hs *Homo sapiens*, Xl *Xenopus laevis*, Dr *Danio rerio*, Bf *Branchiostoma floridae*, Ci *Ciona intestinalis*, Ac *Aplysia californica*, and Lv *Lehmannia valentiana*. The position of primers used for qRT-PCR is this study are shown as qPCR-F (forward primer) and qPCR-R (reverse primer).
(TIF)

Figure S2 **Multiple sequence alignment of translated amino acid sequence of HmHSP90 with other animals.** Black shading indicates completely conserved residues; grey indicates partially conserved residues. Three highly conserved HSP90 family signature sequences are indicated by A, B and C. Hs *Homo sapiens*, Bt *Bos taurus*, Dr *Danio rerio*, Po *Paralichthys olivaceus*, Bf *Branchiostoma floridae*, and Cg *Crassostrea gigas*. The position of primers used for qRT-PCR in this study are shown as qPCR-F (forward primer) and qPCR-R (reverse primer).
(TIF)

Figure S3 **Multiple sequence alignment of NOS derived amino acid sequences.** The locations of amino acid sequence used to design degenerate primers are indicated by DegF1, DegR1, and DegR2. Hs *Homo sapiens*, Xl *Xenopus laevis*, Dr *Danio rerio*, Bf *Branchiostoma floridae*, Ci *Ciona intestinalis*, Ac *Aplysia californica*, and Lv *Lehmannia valentiana*.
(TIF)

Figure S4 **Multiple sequence alignment of HSP90 derived amino acid sequences.** The locations of amino acid sequence used to design degenerate primers are indicated by DegF and DegR. Hs *Homo sapiens*, Bt *Bos taurus*, Dr *Danio rerio*, Po *Paralichthys olivaceus*, Bf *Branchiostoma floridae*, Cg *Crassostrea gigas*.
(TIF)
