# Supplementary figures and images for: Nitric Oxide Acts as a Positive Regulator to Induce Metamorphosis of the Ascidian Herdmania momus
Source: PLoS One. 2013 Sep 3;8(9):e72797. doi: 10.1371/journal.pone.0072797 (PMC3760835; doi:10.1371/journal.pone.0072797)

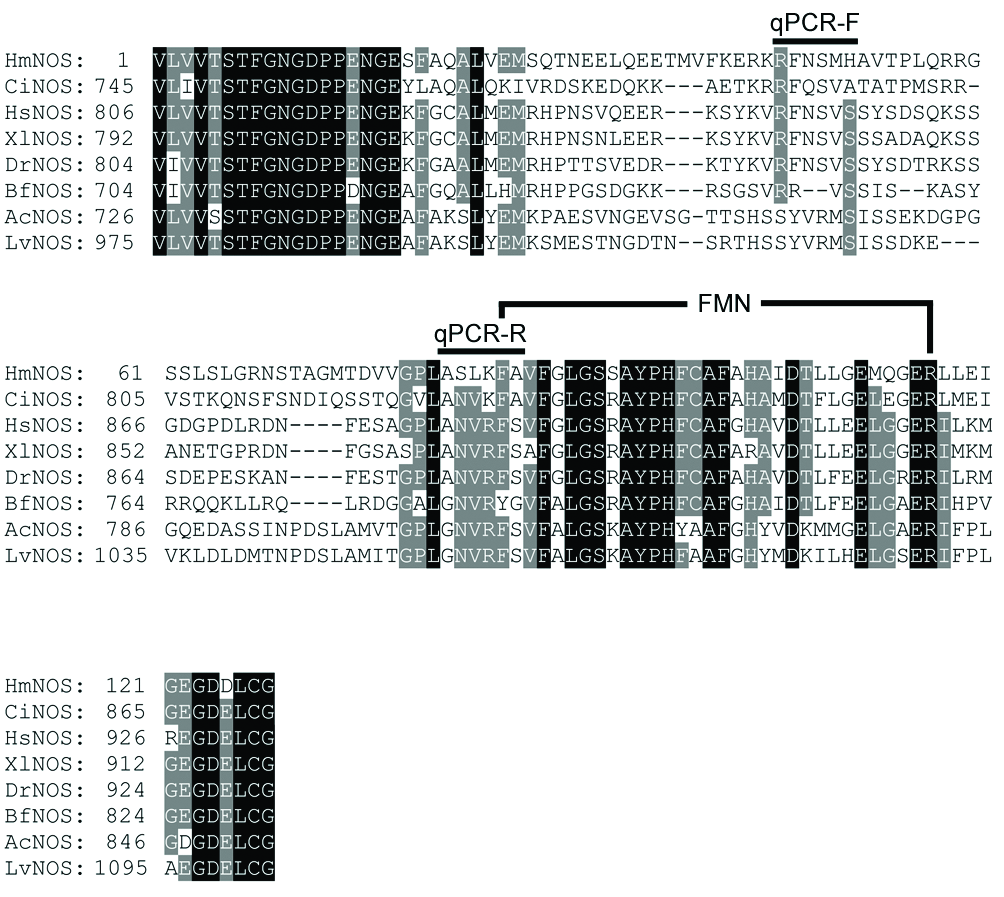

Supplement: Figure S1 — Multiple sequence alignment of translated amino acid sequence of HmNOS with other animals. Black shading indicates completely conserved residues; grey indicates partially conserved residues. FMN delineates the conserved flavin mononucleotide domain. Hm Herdmania momus, Hs Homo sapiens, Xl Xenopus laevis, Dr Danio rerio, Bf Branchiostoma floridae, Ci Ciona intestinalis, Ac Aplysia californica, and Lv Lehmannia valentiana. The position of primers used for qRT-PCR is this study are shown as qPCR-F (forward primer) and qPCR-R (reverse primer). (TIF) [file pone.0072797.s001.tif]

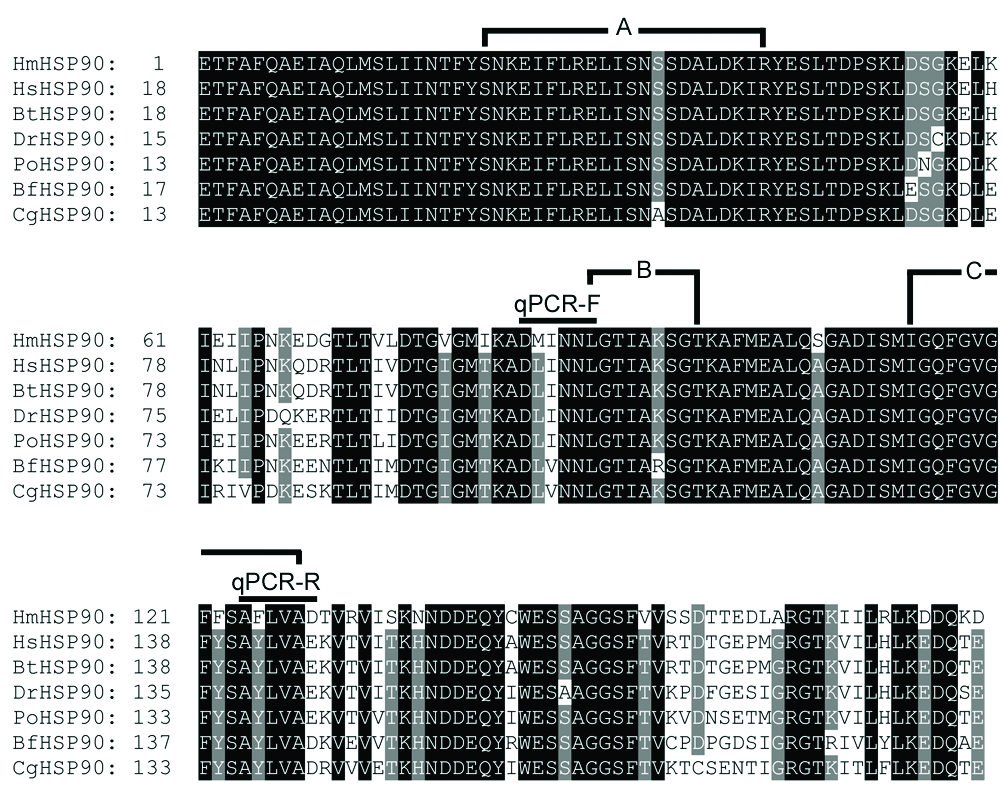

Supplement: Figure S2 — Multiple sequence alignment of translated amino acid sequence of HmHSP90 with other animals. Black shading indicates completely conserved residues; grey indicates partially conserved residues. Three highly conserved HSP90 family signature sequences are indicated by A, B and C. Hs Homo sapiens, Bt Bos taurus, Dr Danio rerio, Po Paralichthys olivaceus, Bf Branchiostoma floridae, and Cg Crassostrea gigas. The position of primers used for qRT-PCR in this study are shown as qPCR-F (forward primer) and qPCR-R (reverse primer). (TIF) [file pone.0072797.s002.tif]

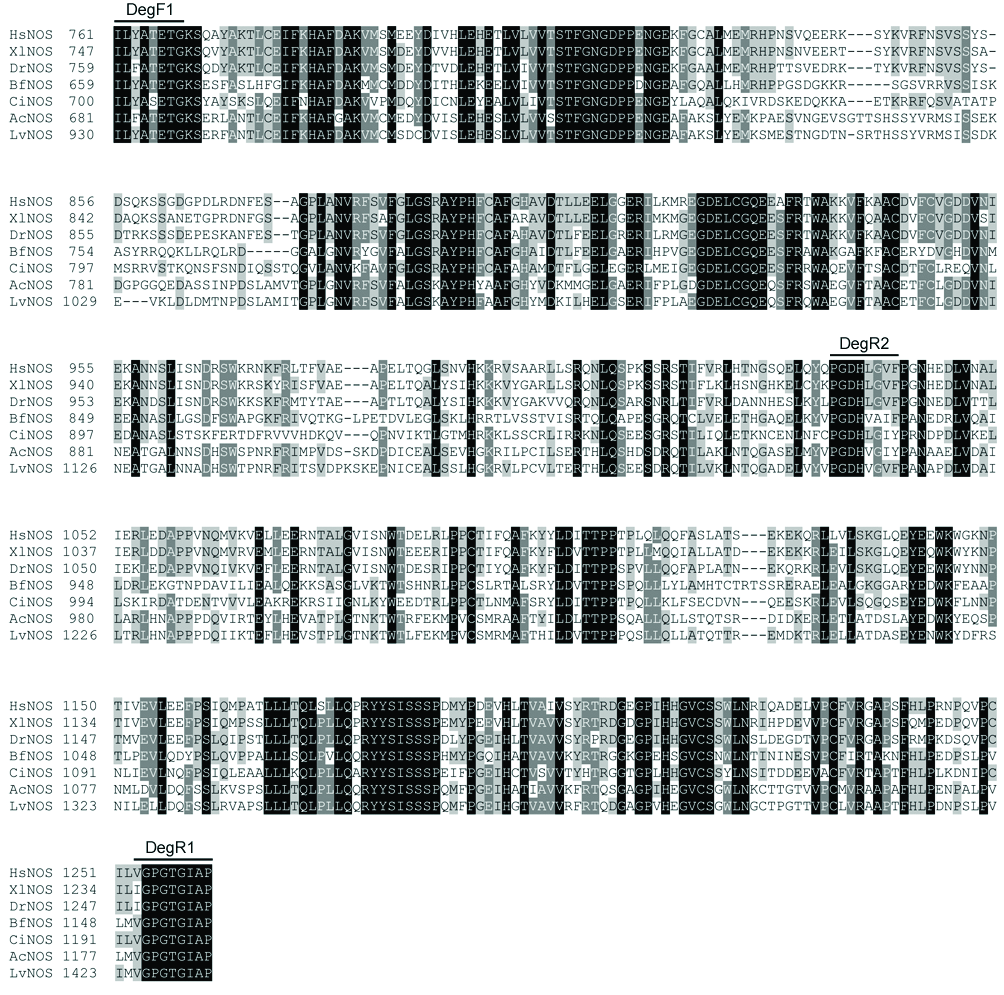

Supplement: Figure S3 — Multiple sequence alignment of NOS derived amino acid sequences. The locations of amino acid sequence used to design degenerate primers are indicated by DegF1, DegR1, and DegR2. Hs Homo sapiens, Xl Xenopus laevis, Dr Danio rerio, Bf Branchiostoma floridae, Ci Ciona intestinalis, Ac Aplysia californica, and Lv Lehmannia valentiana. (TIF) [file pone.0072797.s003.tif]

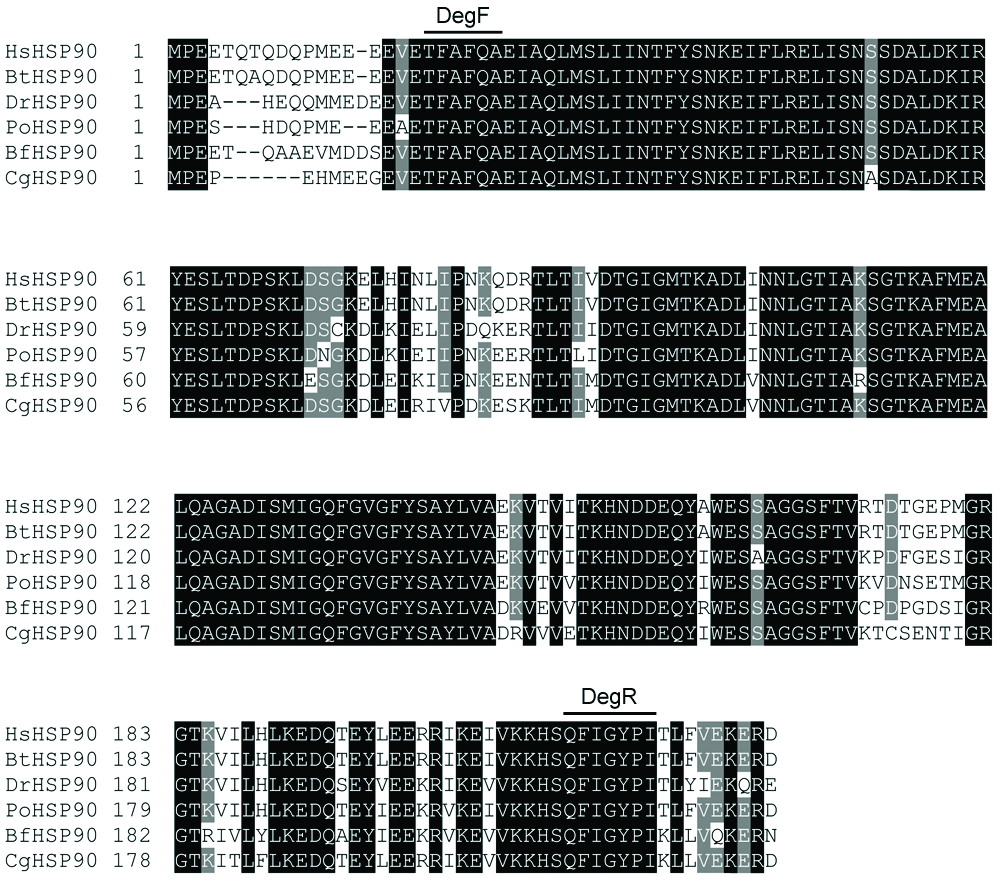

Supplement: Figure S4 — Multiple sequence alignment of HSP90 derived amino acid sequences. The locations of amino acid sequence used to design degenerate primers are indicated by DegF and DegR. Hs Homo sapiens, Bt Bos taurus, Dr Danio rerio, Po Paralichthys olivaceus, Bf Branchiostoma floridae, Cg Crassostrea gigas. (TIF) [file pone.0072797.s004.tif]
